# A retrospective case notes review of the effectiveness and tolerability of metoclopramide in the treatment of clozapine-induced hypersalivation (CIH)

**DOI:** 10.1186/s12888-022-03940-0

**Published:** 2022-04-20

**Authors:** Cecilia Livermore, Hannah White, Loren Bailey, Ian Osborne, Ebenezer Oloyede, Olubanke Dzahini, Eromona Whiskey

**Affiliations:** 1 Pharmacy Department, South London and Maudsley NHS Foundation Trust, Denmark Hill, London, SE5 8AZ UK; 2 Institute of Pharmaceutical Sciences, King’s College London, London, UK; 3 Department of Psychosis Studies, Institute of Psychiatry, Psychology and Neurosciences, King’s College London, London, UK

**Keywords:** Clozapine, Metoclopramide, Hypersalivation, Adverse effect

## Abstract

**Objective:**

The objective of the study is to explore the long-term effectiveness and tolerability of metoclopramide in the treatment of CIH.

**Method:**

This study is a retrospective, observational cohort study of patients prescribed metoclopramide for CIH at the South London & Maudsley (SLaM) NHS Foundation Trust.

**Results:**

Of the 96 patients identified, 14 patients were eligible for inclusion in our study. Five patients continued treatment with a mean duration of 27 months (SD = 17.8), and one patient continued until transfer with a duration of 3 months. Eight patients discontinued treatment after a mean duration of 8 months.

**Conclusion:**

Metoclopramide may be an effective and tolerated drug in CIH, but more data is required to establish its place in the pharmacotherapy of this condition.

## Significant outcomes

- Hypersalivation often persists for many clozapine treated patients but there is no evidence base for long-term treatment of CIH.
- Treatment of CIH should be individualised, but some patients may require a combination of treatments to effectively manage CIH
- Neurological adverse effects of metoclopramide were reported, but evidence is inconclusive.

## Background

Metoclopramide is a substituted benzamide with anti-emetic properties. It is licensed in the UK to treat nausea and vomiting, hiccup, and acute migraine [1]. The anti-emetic effect of metoclopramide appears to be mediated by a combination of antagonism of the D_2_ and 5HT_3_ receptors and agonism of the 5HT_4_ receptors [2]. In addition, metoclopramide stimulates gastric motility and exerts its prokinetic effect by antagonising dopamine mediated relaxation effect on gastrointestinal smooth muscle [3]. Besides the anti-emetic and prokinetic effects of metoclopramide, it is believed that the peripheral activity of the drug can reduce secretion of saliva, and hence it has been considered as a treatment option for clozapine-induced hypersalivation (CIH) also known as sialorrhea [4].

Hypersalivation is a very common adverse effect of clozapine reported by approximately 30–80% of treated patients [5]. It may occur throughout the day, but occurs predominantly at night, and is often reported as bothersome and uncomfortable, and is a potential cause of clozapine discontinuation [6]. This adverse effect is probably dose related and occurs during the early stages of clozapine treatment [7]. CIH may reduce in severity over time, but it may persist. Importantly, CIH and sedation increase the risk of potentially fatal aspiration pneumonia, and hence prompt treatment is necessary [8]. Unfortunately, the evidence base for the management of CIH is poor, and a Cochrane review concluded that there is insufficient data to confidently inform clinical practice [9]. Nevertheless, a more recent systematic review found that metoclopramide with other benzamide derivatives, the anticholinergic propantheline and the antihistamines diphenhydramine and chlorpheniramine demonstrated effectiveness in CIH, but lament the lack of long-term studies [10].

A 3-week, double-blind, placebo-controlled trial found that metoclopramide administered at 10 – 30 mg/day, was a safe and effective agent in the treatment of CIH, demonstrating significant improvement on the Nocturnal Hypersalivation Rating Scale, the Drooling Severity Scale, and the Clinical Global Impression-Improvement scale [4]. While no adverse effects were reported in the study, some of the common side effects that may occur with metoclopramide treatment include diarrhoea, asthenia, drowsiness, hypotension, movement disorders, and parkinsonism [11]. Furthermore, the antidopaminergic action of metoclopramide increases the risk of extrapyramidal side effects, particularly in children and young adults and/or when high doses are used. These effects are generally reversible after stopping metoclopramide, but prolonged treatment may cause tardive dyskinesia, especially in the elderly, which is potentially irreversible [12]. In 2013, the European Medicines Agency (EMA) made recommendations to restrict the use of metoclopramide to a maximum of 30 mg per day for no more than 5 days, due to the high risks of neurological effects [13].

Metoclopramide is now often used in CIH even though there are no long-term safety and efficacy data and in contrast to the EMA recommendations. Randomised Controlled Trials (RCTs) constitute the benchmark for evidence-based medicine. However, RCTs recruit patients based on strict inclusion and exclusion criteria. Whereas, naturalistic observational studies provide evidence for real-world effectiveness. This observational cohort study at the South London and Maudsley NHS Trust is therefore the first attempt to explore the safety and effectiveness of the treatment in a naturalistic setting.

### Aim

The objective of the study is to explore the effectiveness and tolerability of metoclopramide in the treatment of CIH. The primary outcome is continuation of treatment, and the secondary outcome is discontinuation for any reasons.

## Method

This study is a retrospective, observational cohort study. The South London & Maudsley (SLaM) NHS Foundation Trust pharmacy dispensing records and the Zaponex Treatment Access System (ZTAS) database were used to identify all eligible patients. ZTAS is one of the three UK clozapine monitoring registries and is the database where all SLAM clozapine treated patients are registered. All patients co-prescribed clozapine and metoclopramide between 10^th^ April 2016 and 10^th^ March 2021 were eligible for inclusion in the study. The patient electronic health records were used to determine the indication for metoclopramide and scrutinised for indices on efficacy and tolerability of metoclopramide in CIH using free text.

### Variables and measurements

The primary outcome of this study is the continuation of treatment, a widely used outcome measure in observational studies. We collected data on: dose of metoclopramide, duration of treatment, reason for discontinuation, other drugs given concomitantly to treat CIH, and drugs used prior to metoclopramide for CIH. We used the search terms hypersalivation and its synonyms, salivation, dribbling, or drooling. Other search terms used to explore tolerability of metoclopramide included, ‘side effect’, ‘tolerate or tolerability’, ‘extrapyramidal side effects or EPSEs’, ‘tremor’, ‘shaking or shakiness or shake’, ‘Parkinson or parkinsonism’, and synonyms of ‘tardive dyskinesia’, ‘dystonia’, and akathisia’. In addition, data on demographics, diagnosis, and concomitant medications were gathered to describe the cohort.

## Results

A total of 96 patients who received metoclopramide during the study period were identified from our pharmacy dispensing records. Of these, 23 patients were prescribed clozapine. Nine patients prescribed metoclopramide for nausea and vomiting were excluded. Fourteen patients who met inclusion criteria were included, but the other 82 patients who were prescribed metoclopramide for other indications were excluded from the study. The sample comprised 9 males and 5 females with a mean age of 43.8 years [range 22 – 57]. See Table 1. The average daily clozapine dose at metoclopramide initiation was 341 mg [range 225 – 550] and patients had been on clozapine treatment for a mean duration of 79 months [range 3 – 205]. Most patients had a diagnosis of paranoid schizophrenia (*n* = 8), and others had schizoaffective disorder (*n* = 4) and unspecified psychosis (*n* = 2). The dose of metoclopramide prescribed varied from 10 to 30 mg daily. In all patients, at least one medication was tried for CIH prior to metoclopramide. Six of 14 patients received concomitant medication for CIH.

**Table 1:** Clinical characteristics of patients prescribed metoclopramide

Characteristic	Continuers (*n* = 6)	Discontinuers (*n* = 8)	Total (*n* = 14)
Mean age at data collection (years ± SD)	47.8 (± 8.1)	40.8 (± 12.1)	43.8 (± 10.8)
Female gender (%)	2(16.7)	4 (50)	6 (35.7)
Mean clozapine dose (mg ± SD)	362.5 (± 106.9)	325 (± 73.2)	341.1 (± 87.5)
Mean duration of treatment of metoclopramide (years ± SD)	1.9 (± 1.5)	0.7 (± 0.9)	1.2 (± 1.3)
Mean metoclopramide dose (mg ± SD)	21.7 (± 7.5)	18.8 (± 9.9)	20 (± 8.8)
Median metoclopramide dose (IQR)	20 (10)	15 (20)	20 (20)
Metoclopramide monotherapy %	5 (83.3)	3 (37.5)	8 (57.1)
Adverse reactions reported (%)	2 (33.3)	3 (37.5)	5 (35.7)

*IQR* Interquartile range, *SD* Standard deviation

### Treatment continuation

**Fig. 1:**
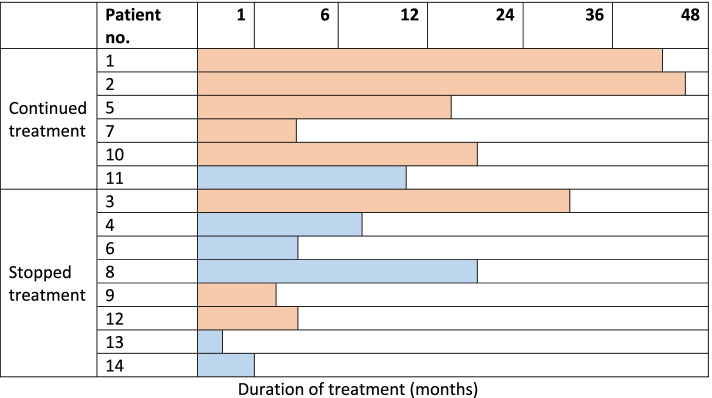
Duration of treatment with metoclopramide in this study sample (*n* = 14). Patients that received metoclopramide as monotherapy for CIH are represented in orange, and those that received concomitant medication for CIH are represented in blue

Six patients continued treatment during the study period with a mean duration of 23 months (including one patient who continued until transfer). Of these, 5 received metoclopramide as monotherapy and one patient as combination therapy. Eight patients stopped treatment with metoclopramide for CIH after a mean duration of 8 months. Five were on combination of medications, and 3 had metoclopramide as monotherapy. See Fig. 1.

### Adverse effects

Five patients in this study had reported adverse effects whilst on metoclopramide for CIH (Table 2). Four patients experienced tremor and one patient had nausea and vomiting. Two patients (no. 6 and 13) discontinued metoclopramide due to adverse effects that had occurred since the initiation of metoclopramide.

**Table 2:** Adverse effects reported by patients in this study

**Patient identifier**	**Adverse effects**	**Onset of symptoms**	**Previous history if EPSE(s)**	**Concomitant medications**	**Did adverse reactions lead to cessation of metoclopramide treatment?**
4	Leg stiffness and tremor	2 months	Yes	Clozapine Sodium valproate Trihexyphenidyl	No
5	Resting tremor in hands and feet Shuffling gait	9 months	Yes	Clozapine Sodium valproate Paracetamol Senna Promethazine Levothyroxine Simvastatin	No
6	Tremor and agitation	2 months	Yes	Clozapine Aripiprazole Sodium valproate Promethazine Lactulose Senna Atropine drops	Yes
10	Resting bilateral tremor	4 months	Yes	Clozapine Aripiprazole Sodium valproate Amisulpride Lithium Diazepam Omeprazole	No
13	Nausea and vomiting	1 day	Yes	Clozapine Sodium valproate	Yes

### Previous medication(s) for CIH

All patients in this study had trialled at least one other medication for CIH prior to using metoclopramide. This includes hyoscine hydrobromide (*n* = 14), pirenzepine (*n* = 7), trihexyphenidyl (*n* = 2), atropine 1% drops (*n* = 2), benzhexol (*n* = 2), benzatropine (*n* = 1), and terazosin (*n* = 1). Six of the 14 patients in this study received metoclopramide as concomitant treatment due to poor response to previous drug, and 7 patients were switched to metoclopramide for CIH as monotherapy. Amongst the group of patients who discontinued metoclopramide (*n* = 8), 6 patients switched to another medication and 4 received the drug that they had previously tried.

### Reasons for discontinuation of metoclopramide

Three patients stopped metoclopramide because it was ineffective, 2 patients discontinued because of adverse effects, in two patients, the reason for discontinuation was unstated in the medical notes and in one instance, due to patient choice.

## Discussion

The exact pathophysiology of CIH is unclear, and several possible mechanisms have been suggested. Clozapine is an agonist at the muscarinic (M_4_) receptor; and an antagonist at the adrenergic receptors (alpha_1_ and alpha_2_), dopamine receptors (D_1_-D_5_), histamine receptor (H_1_), muscarinic receptors (M_1_-M_3_ and M_5_), and serotonin receptors (5-HT_2_ and 5-HT_4_). The alpha_2_ antagonistic and M_4_-muscarinic agonist effects of clozapine can stimulate salivation, however, its anticholinergic effects can reduce the secretion of saliva [14]. Medications with various pharmacological actions have been proposed to manage CIH, such as centrally acting alpha_2_-adrenoceptor agonists (e.g. clonidine, lofexidine), anticholinergics (e.g. hyoscine hydrobromide, atropine 1% drops, pirenzepine), and substitute benzamide derivatives (e.g. amisulpride, sulpiride, metoclopramide) [15–18]. There is no licensed medication for CIH and treatment recommendations are limited by the insufficient available evidence and the risk of bias in the published studies [19].

The mechanism of metoclopramide in reducing hypersalivation is not clearly defined. Substituted benzamide derivatives do not have adrenergic antagonistic, anticholinergic, or adrenomimetic properties [4]. Previous trials with this group of drugs with high selective binding to the dopamine D_2_/D_3_ receptors have shown efficacy in treating CIH [20]. Metoclopramide is a 5-HT_4_ receptor agonist, a mixed 5-HT_3_ receptor antagonist and a dopamine D_2_ receptor antagonist. The antiemetic effect of metoclopramide is presumed to be caused by its D_2_ receptor antagonism in the chemoreceptor trigger zone in the central nervous system, and dry mouth is one of its side effects. The antisalivary effect is presumed to be attributable to the entire substitute benzamide group [21].

The results of this study showed that 6 out of 14 patients (43%) continued treatment with metoclopramide for CIH with a mean duration of 27 months. On the other hand, a total of 8 patients (57%) in this study discontinued metoclopramide with a mean duration of 8 months. It is not known how the continuation rate in our study compares because there are no long-term studies in CIH. In a systematic review of 19 studies in treatment strategies for CIH, the duration of treatment ranged from 10 days to 6 weeks [10].

### Tolerability of metoclopramide

A total of 5 patients (35.7%) in this study reported adverse effects during their treatment with metoclopramide. Four patients experienced tremors between 2–9 months after the initiation of metoclopramide, and one patient discontinued due to this side effect. However, it must be noted that these patients had previously reported EPSE(s) prior to starting metoclopramide. Furthermore, all the patients who reported adverse neurological effects were all prescribed concomitant medications that could cause or exacerbate tremors such as sodium valproate, lithium and other antipsychotics. Nonetheless, metoclopramide is recognised to cause both short and long-term neurological adverse effects, hence the guidance by EMEA in limiting the use to 5 days.

There are currently no guidelines available that recommend a treatment algorithm for CIH including duration of treatment of medications. The most sensible first step in the management of CIH would be to review the dose of clozapine and consider a dose reduction to minimise side effect burden, although this may affect patients’ mental state and the decision should be made by clinicians on an individual basis. The second step would be to consider a trial of medication to manage CIH, such as hyoscine hydrobromide, pirenzepine, and metoclopramide. However, the evidence for the medications used for CIH largely comes from case reports, case series, and several randomised controlled trials. Thus, a direct comparison of the effectiveness between the medications used for CIH cannot be determined due to the lack of good quality evidence [22]. It is notable from our small cohort, many patients continue to suffer from CIH without benefit from various treatments. The mean duration of clozapine treatment at metoclopramide initiation was 6.5 years and all patients had been tried on at least one medication prior to metoclopramide. duration.

Despite the potential neurological adverse effect of metoclopramide, it is an agent that warrants further investigation, not least because, many of the agents used in CIH can cause constipation, a potentially fatal complication of clozapine use. Where possible, agents that cause constipation should best be avoided with clozapine. Metoclopramide is an agonist at 5HT_4_ whereas clozapine is a potent antagonist at this receptor site. The activity of clozapine at this receptor site contributes to the burden of constipation. It is therefore a potential benefit for using one agent to mitigate both CIH and constipation.

Our study is a retrospective case notes review which has some limitations: small sample size, lack of measurements of serum clozapine levels and a lack of accurate and objective measurement of the effectiveness and tolerability of metoclopramide as no hypersalivation rating scales were used. While, real-world persistence has been suggested to be a proxy for treatment effectiveness and adherence, it is plausible that some continued treatment despite limited efficacy. The response to treatment was mainly determined by observation of drooling and patient experience as recorded in the medical notes. Moreover, the response to metoclopramide may be influenced by the dose of clozapine. We did not track the changes in clozapine dose during the study period. Finally, the lack of systematic reporting of adverse events means our results need to be interpreted cautiously.

## Conclusion

Metoclopramide may be an effective and tolerated drug in CIH. Larger, well-designed, trials with bigger sample sizes and accurate measurements of efficacy and tolerability of metoclopramide are required to establish its place in the pharmacotherapy of CIH.

## Data Availability

The data that support the findings of this study are available from the corresponding author upon reasonable request.

## References

[1] British National Formulary (2021). Metoclopramide hydrochloride. [online] NICE. Available at: https://bnf.nice.org.uk/drug/metoclopramide-hydrochloride.html [Accessed 19 May 2021].

[2] Henzi I, Walder B, Tramer MR (1999). Metoclopramide in the prevention of postoperative nausea and vomiting: a quantitative systematic review of randomized, placebo-controlled studies. Br J Anaesth.

[3] Martindale (2021). Digital Medicines Information Suite - metoclopramide. [online] MedicinesComplete. Available at: https://www.medicinescomplete.com/#/content/martindale/11464-n#content%2Fmartindale%2F11464-n%2311464-n [Accessed 19 May 2021].

[4] Kreinin A, Miodownik C, Mirkin V, Gaiduk Y, Yankovsky Y, Bersudsky Y, Lerner PP, Bergman J, Lerner V (2016). Double-Blind, Randomized, Placebo-Controlled Trial of Metoclopramide for Hypersalivation Associated With Clozapine. J Clin Psychopharmacol.

[5] EMC (2020). Zaponex 100 mg Tablets - Summary of Product Characteristics (SmPC) - (emc). [online] www.medicines.org.uk. Available at: https://www.medicines.org.uk/emc/product/7715/smpc#UNDESIRABLE_EFFECTS [Accessed 19 May 2021].

[6] Legge SE, Hamshere M, Hayes RD, Downs J, O'Donovan MC, Owen MJ, MacCabe JH (2016). Reasons for discontinuing clozapine: a cohort study of patients commencing treatment. Schizophr Res.

[7] Praharaj SK, Arora M, Gandotra S (2006). Clozapine-induced sialorrhea: pathophysiology and management strategies. Psychopharmacology.

[8] NICE (2013). Intervention and alternatives | Hypersalivation: oral glycopyrronium bromide | Advice | NICE. [online] www.nice.org.uk. Available at: https://www.nice.org.uk/advice/esuom15/chapter/intervention-and-alternatives [Accessed 19 May 2021].

[9] Syed R, Au K, Cahill C, Duggan L, He Y, Udu V, Xia J. Pharmacological interventions for clozapine-induced hypersalivation. Cochrane Database Syst Rev. 2008;(3):CD005579. 10.1002/14651858.CD005579.pub2. 10.1002/14651858.CD005579.pub2 PMC4160791 18646130

[10] Chen SY, Ravindran G, Zhang Q, Kisely S, Siskind D (2019). Treatment strategies for clozapine-induced sialorrhea: a systematic review and meta-analysis. CNS Drugs.

[11] EMC (2020). Metoclopramide 10mg Tablets - Summary of Product Characteristics (SmPC) - (emc). [online] www.medicines.org.uk. Available at: https://www.medicines.org.uk/emc/product/5880/smpc.

[12] Rao AS, Camilleri M (2010). metoclopramide and tardive dyskinesia. Aliment Pharmacol Ther.

[13] EMA (2013). European Medicines Agency recommends changes to the use of metoclopramide.

[14] Rabinowitz T, Frankenburg FR, Centorrino F, Kando J (1996). The effect of clozapine on saliva flow rate: A pilot study. Biol Psychiat.

[15] Van der Poorten T (2019). The sublingual use of atropine in the treatment of clozapine-induced sialorrhea: a systematic review. Clin Case Rep.

[16] McKane JP (2001). Hyoscine patches in clozapine-induced hypersalivation. Psychiatr Bull.

[17] Fritze J (1995). Pirenzepine for clozapine-induced hypersalivation. Lancet.

[18] Duggal HS (2007). Glycopyrrolate for clozapine-induced sialorrhea. Prog Neuropsychopharmacol Boil Psychiatry.

[19] Sockalingam S (2009). Review: Insufficient evidence to guide use of drugs for clozapine induced hypersalivation. Evid Based Ment Health.

[20] Kreinin A, Novitski D, Weizman A (2006). Amisulpride treatment of clozapine-induced hypersalivation in schizophrenia patients: a randomized, double-blind, placebo-controlled cross-over study. Int Clin Psychopharmacol.

[21] Beersheva Mental Health Center (2014). Clinical Trial on Clozapine-induced Hypersalivation: Metoclopramide, placebo - Clinical Trials Registry - ICH GCP. [online] ichgcp.net. Available at: https://ichgcp.net/clinical-trials-registry/NCT02222220 [Accessed 19 May 2021].

[22] Gupta S, Khastgir U, Croft M, Roshny S (2019). Management of clozapine-induced sialorrhoea. BJPsych. Advances.

